# Poverty and Covid-19: Rates of Incidence and Deaths in the United States During the First 10 Weeks of the Pandemic

**DOI:** 10.3389/fsoc.2020.00047

**Published:** 2020-06-15

**Authors:** W. Holmes Finch, Maria E. Hernández Finch

**Affiliations:** Department of Educational Psychology, Ball State University, Muncie, IN, United States

**Keywords:** COVID-19, poverty, inequality, testing, coronavirus (2019-nCoV)

## Abstract

The Covid-19 pandemic in the winter and spring of 2020 represents a major challenge to the world health care system that has not been seen perhaps since the influenza pandemic in 1918. The virus has spread across the world, claiming lives on all continents with the exception of Antarctica. Since its arrival in the United States, attention has been paid to how Covid-19 cases and deaths have been distributed across varying socioeconomic and ethnic groups. The goal of this study was to examine this issue during the early weeks of the pandemic, with the hope of shedding some light on how the number of cases and the number of deaths were, or were not related to poverty. Results of this study revealed that during the early weeks of the pandemic more disadvantaged counties in the United States had a larger number of confirmed Covid-19 cases, but that over time this trend changed so that by the beginning of April, 2020 more affluent counties had more confirmed cases of the virus. The number of deaths due to Covid-19 were associated with poorer and more urban counties. Discussion of these results focuses on the possibility that testing for the virus was less available in more disadvantaged counties later in the pandemic than was the case earlier, as the result of an overall lack of adequate testing resources across the nation.

The emergence of the Covid-19 virus across the world, beginning in late 2019, has put the health care systems of many nations under a great deal of stress. Indeed, in some countries, such as Italy and Spain, this stress has brought health care to the breaking point, resulting in a large number of deaths. In other nations, including Singapore, Korea, and Germany, the number of per capita deaths has been very low in comparison. In each of these countries, the widespread availability of testing, followed by contact tracing has been credited with the relatively low mortality figures, and slowed spread of the virus (National Public Radio, 2020).

As has been reported in a number of sources, the United States has produced an uneven response to the emergence of Covid-19, and has suffered from a lack of testing resources (ProPublica, 2020). As such, the large scale testing and tracing responses seen in Singapore, Korea, and Germany have not been possible in the U.S., leading to a strategy relying heavily on physical distancing and lockdowns to slow the spread of the virus. As of this writing, the extent to which this strategy has been successful is not yet clear, and may not be totally understood for quite some time.

One persistent set of reports in the popular media has focused on the apparently outsized toll that Covid-19 is taking in communities of color, and among under-resourced individuals living in urban areas (Washington Post, 2020). These reports suggest that such communities may be suffering more cases and particularly deaths than would be expected based upon their share in the general population. Such reports are especially concerning given that under-resourced communities frequently have less access to high quality health care, and suffer from more illnesses that are associated with high mortality, such as diabetes, heart disease, and pulmonary issues (Link and Phelan, 1995; Braveman et al., 2005; Lutfey and Freese, 2005; Adler and Rehkopf, 2008; Elo, 2009; Williams et al., 2010; Oates et al., 2017). Therefore, a higher presence of Covid-19 within this population could be particularly disastrous in terms of mortality. Research examining the relationship between poverty and influenza has demonstrated that vaccinations in particular are less available to residents of poorer counties within the United States, than those who live in more affluent areas (Lee et al., 2011). Please note that throughout the manuscript we use the terms poverty, poor, under-resourced, and low income to refer to communities who lack crucial economic resources.

In addition to problems associated with a greater hazard of death due to Covid-19, individuals living in lower income communities may also have less access to high quality health care (Lorant et al., 2002; Shi and Steven, 2005; James et al., 2008). Furthermore, a U.S. Bureau of Labor Statistics report from 2018 (U.S. Bureau of Labor Statistics, 2018) indicates that workers with lower levels of education are less likely to work from home, suggesting that they therefore may also be less able to physically distance than those with higher levels of education. In turn, these individuals may be faced with the choice between staying home and not getting paid, or going to work and increasing their risk of becoming infected with the virus. In addition, these people may also have less access to testing and treatment resources, if experience with influenza is any guide (Ompad et al., 2007; Logan, 2009). Considering all of these issues together, Covid-19 presents under-resourced Americans with a set of unique and potentially very dangerous challenges. In addition, policy makers who are struggling to deal with the effects of the pandemic across the nation at large may not have the resources to focus on this particularly vulnerable portion of the population.

## Study Purpose

Given the issues cited above, particularly the potentially high degree of vulnerability to the effects of the Covid-19 pandemic for people living with poverty, the purpose of this study was to examine the relationship between poverty and the number of confirmed Covid-19 cases and deaths early in the pandemic in the United States. More specifically, poverty was defined using an index from the University of Michigan (https://poverty.umich.edu/about/) that incorporates a variety of variables including social mobility, life expectancy, percent of residents living both below the poverty line and in deep poverty, and the percent of low birth weights. Our goal, therefore, was to investigate associations between the overall index and Covid-19 incidence and death rates, as well as the relationships between these outcomes and the individual variables constituting the index. There have been a number of media reports regarding the apparently disproportionate impact of the virus on people of color who live in relatively poorer urban enclaves across the nation. Therefore, it was of interest to ascertain the extent to which this apparent pattern was true in the very early stages of the pandemic in the U.S., and whether that relationship changed over time. Having insights into these issues will help policy makers deal with the current health crisis, and prepare for the next one.

## Methods

A set of statistical models were used to address the purposes of this study as outlined above. Data from two sources, which are described below, were put together in order to allow for the examination of relationships between relative county poverty and the number of confirmed Covid-19 cases and deaths.

### Data Sources

Two data sources were used in the current study. In order to obtain the numbers of confirmed Covid-19 cases and deaths, the dataset provided by the New York Times was used. The data were downloaded from https://github.com/nytimes/covid-19-data on April 3, 2020. A full description of the dataset appears on the data website, with a brief description here. The data were collected beginning in late January, 2020 with the first case in the set being January 21, 2020. As described on the website, the data were collected from state and local health departments. For the purposes of this study, data broken down at the county level were used. The FIPS code for each county was included in the dataset, which allowed for it to be merged with other datasets that also include this county identifier.

The poverty data used in this study came from the Poverty Solutions Initiative (PSI) at the University of Michigan (https://poverty.umich.edu/about/). Specifically, the Index of Deep Disadvantage, hereafter referred to simply as the poverty index, or index, served as the primary independent variable. It is described below in greater detail. The poverty index data for each county in the United States is available, along with the values of the constituent variables making it up, and the county FIPS code. This latter value allowed for these data to be merged with the NY Times Covid-19 data. The developers of the index provide a full description of their data sources (https://poverty.umich.edu/files/2020/01/IDD-Technical-documentation-1.pdf). The individual variables used to develop this index, in addition to the index itself, are described below.

### Variables

The outcome variables of interest in this study were the cumulative numbers of confirmed Covid-19 cases and deaths for each county in the United States. As noted above, these were obtained by the NY Times from state and local health authorities. Therefore, when reading the results presented below it is important to keep in mind that only cases and deaths that have been confirmed by state and local health authorities are included here. Thus, as discussed at the end of the manuscript, the issue of testing availability and access are important in considering the findings.

The poverty index was developed by researchers in the PSI using principal components analysis (PCA). More specifically, the index was the first principal component obtained using PCA involving five features that have been demonstrated to be associated with poverty and disadvantage (Robles et al., 2019). The researchers reported that this first component accounted for more than 60% of the variance in the set of variables. The weights obtained from the PCA were then applied to the set of constituent variables in order to obtain an index score for each community. After obtaining the index scores, the researchers undertook sensitivity analyses in order to ensure that the index was, in fact, reflecting relative disadvantage as was its intent. The results of these sensitivity analyses did indeed support the validity of the index, as reported in Robles, Simington, and Shaefer (https://poverty.umich.edu/files/2020/01/IDD-Technical-documentation-1.pdf). The index is scaled such that higher values indicate a higher degree of *advantage*; i.e., relatively more prosperous communities. Thus, lower scores were associated with communities experiencing greater economic disadvantage.

Several variables were used in constructing the poverty index. These include the Chetty and Hendren (2018) estimate of social mobility, life expectancy, percent of residents living below the poverty line, percent of residents living in deep poverty, and the percent of low birth weights. In addition, the PSI also collected other variables that might be associated with poverty, including whether the community was urban or not, and percent of residents with less than a high school diploma. Communities were defined as urban based on a definition used by the National Center for Health Statistics, and appearing at this website: https://www.cdc.gov/nchs/data_access/urban_rural.htm. Specifically, urban counties contained a metropolitan statistical area (MSA) of 1 million or more individuals, or that have the entire population contained within the largest principal city of the MSA, or contain at least 250,000 in habitants of any principal city of the MSA. In addition, urban counties were also defined as those with a population of 1,000,000 or higher but which did not meet the aforementioned standards, or those with MSAs of 250,000–999,999.

### Data Analysis

In order to address the study goals, two statistical models were employed using SAS version 9.4 (SAS Institue, 2019). In order to assess the relationship between poverty and the number of confirmed Covid-19 cases, multiple regression was used, with the independent variables being the date, the poverty index, and the interaction of the two variables. Both date and the poverty index were centered prior to estimation of the model. It should be noted that SAS stores dates as the number of days before or after 1/1/1960. The mean of the dates for this sample were calculated and used to center each of the values used in the analysis. The dependent variable in this case was the number of confirmed Covid-19 cases for the date in question. Analyses were conducted using both the raw frequency, and the frequency standardized to county population, with results of the two approaches being very similar to one another. Results for the raw frequencies appear in the Results section. In order to follow up a statistically significant result for the interaction between date and the poverty index, simple slopes relating the index to the number of cases were calculated at selected dates. In addition to investigating the relationship between the number of cases and the index, regression was also used to examine the extent to which the individual variables that make up the index were associated with the number of Covid-19 cases. As for the overall index, these regression models included the main effects for date and the variable, as well as the interaction between the two. And, as with the index, variables were centered prior to the calculation of the interaction term. Given the high collinearity among the individual variables (VIF>5 for several variables), these regression models were fit for each variable individually. In order to ensure that the models properly accounted for the autocorrelation in the outcome variables, The Durbin-Watson statistic was calculated for the model residuals, and no autocorrelation was found to be present. In addition to collinearity and residual autocorrelation, the assumptions of normality and homogeneity of variance were also assessed and found to be satisfied.

The second primary analysis in this study involved treating the number of confirmed deaths due to Covid-19 as the response variable, with date, index, and their interaction serving as the independent variables. Given the relatively low number of deaths relative to the number of counties, particularly early in the pandemic, a Poisson regression model was fit to the data. As with the incidence data, models using both the raw death counts and the death counts standardized by county population were fit. Results for the two ways of expressing the death rate were extremely similar, and thus the raw death counts are reported in the Results section. The appropriateness of this model was assessed with the deviance statistic, which was found to support the use of Poisson regression, as described below. The value was close to 1, and the Chi-square test comparing it to 1 was not statistically significant. Thus, the data was found not to be either under or overdispersed. As with the regression model for number of cases, a statistically significant interaction result was followed up by simple slopes at selected dates. The Poisson regression model was also used to assess the relationships between individual measures of poverty and the number of confirmed deaths due to Covid-19.

## Results

### Descriptive Statistics

**Figure 1:**
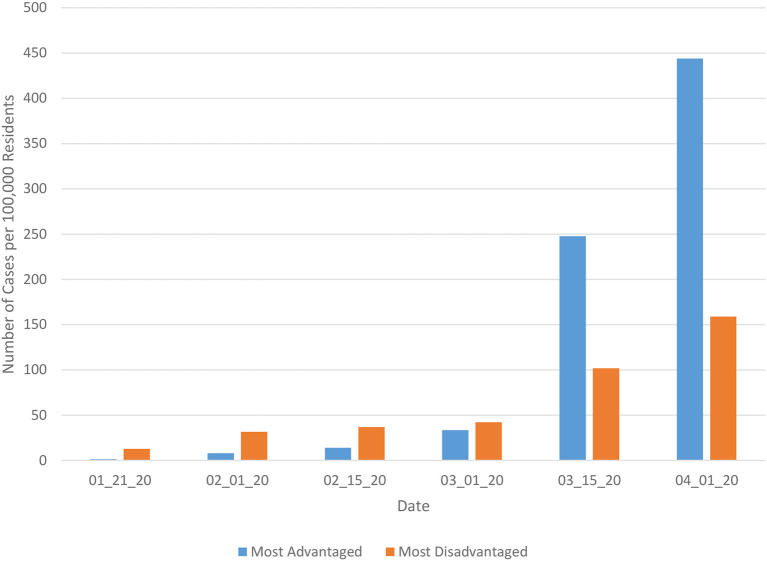
Number of Covid-19 cases per 100,000 residents by date for most advantaged and most disadvantaged counties.

A total of 2,853 of the 3,007 counties in the United States were included in the current analysis. Data used in this study were collected over a 71 day period, from January 21, 2020 through April 1, 2020. The means and standard deviations of the poverty index, as well as the constituent variables used to calculate it appear in Table 1. The number of cases per 100,000 residents in each county appears in Figure 1, separated by the 100 most advantaged and 100 most disadvantaged counties, based on the index value. These results reveal that early in the pandemic, the number of cases per 100,000 residents were higher in the 100 most disadvantaged counties. However, from the middle of March forward, the number of cases per 100,000 residents was larger in the 100 most advantaged counties, and the difference between the two increased over time.

**Table 1:** Mean and standard deviation for poverty index, and its constituent variables.

**Variable**	**Mean**	**Standard deviation**
Index	0.11	1.72
Mobility	44.34	4.49
Percent below poverty	15.24	6.19
Percent in deep poverty	6.70	3.19
Life expectancy	78.08	2.95
Percent low birth weight	8.11	1.89
Percent urban	59.11	49.17
Percent less than high school	12.75	5.85

### Covid-19 Cases

As noted in the Methods section, a mixed effects linear modeling approach was applied to the number of Covid-19 cases reported by U.S. counties. Three models were fit to the data, including a null model with no predictors, a model in which date is the only predictor, and a third model in which date, the poverty index, and the interaction of the two variables serves as a predictor. Based on results from the null model, the intraclass correlation (ICC) for the county effect was 0.169, meaning that ~16.9% of the variation in the number of cases can be accounted for by the county. The results in Table 2 indicate that the model including both date and the poverty index yielded the best fit to the data, based on AIC, AICC, and BIC.

**Table 2:** Mixed effects linear model fit statistics for number of Covid-19 cases.

**Model**	**AIC**	**AICC**	**BIC**
Null	371658.2	371658.2	371670.6
Date only	370205.4	370205.4	370217.8
Date and poverty index	370118.6	370118.6	370131.0

The coefficients for the fixed effects of the date, index, and their interaction appear in Table 3, along with the intercept. Note that the two variables were centered prior to the fitting of the model. Date was positively associated with the number of cases, and there was a statistically significant interaction between the date and poverty index. Therefore, the primary focus will be given to interpreting the nature of this interaction, rather than on the main effects.

**Table 3:** Fixed effects results for optimal number of cases linear mixed effects model.

**Effect**	**Coefficient**	**Standard error**	**Confidence interval**
Intercept	−1.35	2.56	−6.37, 3.67
Date	7.61	0.21	7.19, 8.03
Poverty index	0.93	1.49	−1.98, 3.85
Date*poverty index	1.22	0.14	0.94, 1.50

Table 4 includes the simple slope estimates relating the poverty index to the number of cases at specific dates. For the first three dates, the relationship between the poverty index and the number of cases was negative, indicating that counties with greater levels of reported poverty had a larger number of confirmed Covid-19 cases. However, by April 1, 2020, the relationship between these two variables was positive, so that relatively more affluent counties had a larger number of confirmed cases. It is also important to note that for the earlier dates, the number of confirmed cases overall was relatively small.

**Table 4:** Mixed effects linear model simple slopes, standard errors, *t*, and *p*-values, for the relationship between poverty index and the number of cases^*^.

**Date**	**Coefficient**	**Standard error**	***t***	***p*-value**	**Cumulative cases**
February 15, 2020	−45.11	6.26	−7.20	<0.0001	179
March 1, 2020	−26.86	4.24	−6.33	<0.0001	425
March 15, 2020	−9.82	2.54	−3.86	0.0001	3,613
April 1, 2020	10.87	1.79	6.09	<0.0001	216,622

* *For all coefficients DF = 23,586*.

In order to understand which aspects of poverty were associated with the number of cases, certain of the constituent variables making up the index were each included as an independent variable in a mixed effects model with cases as the dependent variable. Specifically, the percent of individuals living below poverty, the percent of residents in deep poverty, social mobility, the percent of residents with less than a high school diploma, whether a county was classified as urban, life expectancy, and the percent of low birth weights were all examined in this follow up analysis. As was the case for the poverty index, the main effects of date, the specific variable, and the interaction of the two were included in a mixed effects model with a random intercept. Each poverty variable was analyzed individually in order to avoid the possibility of collinearity among them.

Table 5 includes the coefficient, standard error, and 95% confidence intervals for the main effects and interactions of each poverty variable with number of cases as the outcome. These results reveal that with respect to the number of Covid-19 cases, there was a statistically significant interaction of date with percent of residents living in poverty, percent living in deep poverty, social mobility, whether the county was urban, life expectancy, and percent low birth weights. Given the statistical significance of the interactions between date and several of these variables, the simple slopes were examined, as with the overall poverty index. The one exception to this outcome was for the percent of county residents who had less than a high school diploma, for which the interaction with date was not statistically significant, but the main effect was. The negative coefficient indicates that counties with a higher percent of residents having less than a high school diploma had fewer cases.

**Table 5:** Linear mixed effects model fixed effects results of specific poverty variables with number of Covid-19 cases.

**Effect**	**Coefficient**	**Standard error**	**Confidence interval**
**Percent of residents living in poverty**			
Intercept	−0.69	2.58	−5.74, 4.37
Date	7.27	0.22	6.83, 7.70
Percent in poverty	3.54	2.73	−1.81, 8.88
Date*percent in poverty	2.92	0.26	2.40, 3.45
**Percent of residents Living In Deep Poverty**			
Intercept	−2.63	2.56	−7.65, 2.39
Date	7.68	0.21	7.26, 8.10
Percent in deep poverty	0.71	2.74	−4.66, 6.09
Date*percent in deep poverty	2.32	0.27	1.79, 2.85
**Social mobility index**			
Intercept	−3.84	2.63	−9.01, 1.32
Date	7.79	0.22	7.36, 8.23
Social mobility	−7.34	3.05	−13.31, −1.37
Date*social mobility	−1.28	0.30	−1.86, −0.70
**Percent of residents with less than high school diploma**			
Intercept	24.27	6.14	12.23, 36.30
Date	7.93	0.53	6.89, 8.97
< High school	−2.14	0.43	−2.98, −1.30
Date* < high school	0.02	0.04	−0.07, 0.10
**Urban**			
Intercept	0.56	4.07	−7.41, 8.53
Date	0.54	0.47	−0.37, 1.46
Urban	35.96	5.53	25.12, 46.80
Date*urban	10.48	0.53	9.45, 11.51
**Life expectancy**			
Intercept	7.07	2.62	1.92, 12.21
Date	5.53	0.25	5.05, 6.02
Life expectancy	10.27	2.61	5.14, 15.39
Date*life expectancy	4.29	0.22	3.86, 4.71
**Low birth weight**			
Intercept	−2.96	2.63	−8.12, 2.10
Date	8.54	0.22	8.11, 8.97
Percent low birth weight	6.35	2.79	0.89, 11.82
Date*low birth weight	−1.33	0.27	−1.86, −0.80

The simple slopes for those poverty variables that had a statistically significant interaction with date appear in Table 6 (note that because the percent of residents with less than a high school diploma did not interact with date, it does not appear in in the table). For the percent of residents living in poverty and those living in deep poverty, the interaction with time was very similar to what is reported above for the poverty index. Namely, early in the pandemic, poorer counties had a higher rate of confirmed cases than did relatively less poor areas. Furthermore, areas with higher levels of social mobility had relatively fewer cases later in the pandemic, whereas there was not a statistically significant relationship between social mobility and the number of confirmed cases early in the pandemic. This pattern regarding counties with higher rates of poverty having more confirmed cases early in the pandemic can also be seen in Table 7. The percent of individuals in population-dense urban counties, as well as those living in poverty, deep poverty, and with less than a high school diploma for counties with confirmed cases all declined over time. In addition the social mobility index increased in value over this period, indicating that counties with confirmed cases later in the time period exhibited more social mobility than did those counties with confirmed cases early in the pandemic, vs. later. Finally, counties with lower life expectancies had more identified cases earlier in the pandemic, whereas by April 1, 2020 counties with longer life expectancies had more cases. Similarly, counties with a percentage of low birth weights also had a larger number of identified cases early in the pandemic, and by April 1, 2020 there was not a statistically significant relationship between percent low birth weight and the number of cases.

**Table 6:** Simple slopes, standard errors, *t*, and *p*-values, for the relationship between poverty variables and the number of confirmed Covid-19 cases (cumulative cases by date appear in Table 3).

**Date**	**Coefficient**	**Standard error**	***t***	***p*-value**
**Percent living in poverty**				
February 15, 2020	98.40	16.14	−6.10	<0.0001
March 1, 2020	54.62	13.06	−4.18	<0.0001
March 15, 2020	13.74	10.71	−1.28	0.1994
April 1, 2020	−35.88	9.13	3.93	<0.0001
**Percent living in deep poverty**				
February 15, 2020	85.55	16.51	−5.18	<0.0001
March 1, 2020	50.78	13.29	−3.82	<0.0001
March 15, 2020	18.34	10.83	−1.69	0.0902
April 1, 2020	−21.06	9.15	2.30	0.0214
**Social mobility index**				
February 15, 2020	22.69	18.73	1.21	0.2259
March 1, 2020	3.48	15.10	0.23	0.8176
March 15, 2020	−14.44	12.20	−1.18	0.2364
April 1, 2020	−36.21	9.96	−3.64	0.0003
**Urban**				
February 15, 2020	894.82	208.29	4.34	<0.0001
March 1, 2020	1051.97	203.16	5.18	<0.0001
March 15, 2020	1198.66	200.46	5.98	<0.0001
April 1, 2020	1376.77	197.51	6.97	<0.0001
**Life expectancy**				
February 15, 2020	−126.56	13.87	−9.13	<0.0001
March 1, 2020	−62.26	11.58	−5.38	<0.0001
March 15, 2020	−2.25	9.93	−0.23	0.8212
April 1, 2020	70.63	8.96	7.88	<0.0001
**Percent low birth weight**				
February 15, 2020	72.97	16.40	4.45	<0.0001
March 1, 2020	53.04	13.27	4.00	<0.0001
March 15, 2020	34.44	10.91	3.16	0.0016
April 1, 2020	11.86	9.40	1.26	0.21

**Table 7:** Percent urban population, living in poverty, deep poverty, and with less than a high school education, and social mobility for counties with confirmed Covid-19 cases by date.

**Dates**	**Percent urban**	**Percent living in poverty**	**Percent living in deep poverty**	**Percent with less than high school**	**Social mobility**
01/21/20–02/15/20	100.0	15.1	6.8	15.6	43.1
02/15/20–03/01/20	95.2	14.7	6.7	13.9	43.4
03/02/20–03/15/20	86.6	13.0	5.8	10.9	44.0
03/16/20–04/01/20	56.0	12.3	5.3	10.5	44.4

### Covid-19 Deaths

As with the number of Covid-19 cases, a mixed effects model was used to assess the relationships between time, poverty index scores, and their interaction with the number of deaths due to the virus. Given the relatively small number of deaths early in the pandemic, a Poisson regression model was used, rather than a linear mixed model as for the cases. The possibility of overdispersion was assessed using the dispersion parameter and associated Chi-square test of the null that this value was 1 in the population. The dispersion parameter was 1.23, and the Chi-square test was not statistically significant (*p* = 0.96), indicating that overdispersion of the death counts was not an issue.

The AIC and BIC statistics for the null, date only, and date with poverty index and interaction models appear in Table 8. The model including both date and poverty, along with their interaction, yielded the best fit to the data. Based on the results for this model (Table 9), it appears that date, the poverty index, and the interaction were all associated with the number of deaths. The simple slopes for the poverty index at selected dates appear in Table 10. Given the very low number of deaths early in the pandemic, two dates from later in the study period were selected for use here. At the earlier date, there was not a statistically significant relationship between the poverty index value and the number of deaths. However, by April 1, 2020, there was a negative association between the two variables, indicating that for counties with a higher index value (i.e., more prosperous counties) there were fewer deaths than was the case for counties with lower index scores. In other words, the death rate was higher for relatively poorer counties.

**Table 8:** Mixed effects Poisson regression model fit statistics for number of number of Covid-19 deaths.

**Model**	**AIC**	**BIC**
Null	43570.5	43586.8
Date only	20692.9	20717.4
Date and poverty index	19830.7	19871.5

**Table 9:** Mixed effects Poisson regression model fixed effects coefficients, standard errors, and 95% confidence interval for optimal number of deaths model.

**Effect**	**Coefficient**	**Standard error**	**Confidence interval**
Intercept	−8.582	0.223	−9.026, −8.134
Date	0.223	0.002	0.216, 0.224
Poverty index	0.208	0.067	0.076, 0.346
Date*poverty index	−0.30	0.10	−0.20, −0.40

**Table 10:** Mixed effects Poisson regression model simple slopes, standard errors, *t*, and *p*-values, for the relationship between poverty index and the number of deaths.

**Date**	**Coefficient**	**Standard error**	***t***	***p*-value**	**Cumulative deaths**
March 22, 2020	0.81	0.49	1.64	0.0632	504
April 1, 2020	−2.19	0.45	−4.81	0.0003	4,778

In order to more fully understand the nature of the significant interaction described above, several of the constituent variables were included as independent variables along with date in Poisson regression models. The coefficients for these models appear in Table 11, and demonstrate that there was a statistically significant interaction between percent of residents living in poverty, percent living in deep poverty, urban location, and percent low birth weight with date, indicating that their relationships with the number of deaths attributed to Covid-19 changed over time. The simple slopes for these variables at the two selected times appear in Table 12. The coefficients show that the number of deaths increased over time more quickly in those counties with higher percentages of residents living in poverty, and deep poverty, those living in urban areas, and in counties with a higher proportion of babies born at low weight. This result is consonant with the higher rates of confirmed Covid-19 cases in relatively poorer and more urban areas earlier in the pandemic, so that by the end of the study period the disease had progressed for some individuals to the point of death.

**Table 11:** Mixed effects Poisson regression model fixed effects coefficients, standard errors, and 95% confidence interval for main effects and interactions of specific poverty variables for number of deaths.

**Effect**	**Coefficient**	**Standard error**	***t***	***p*-value**
**Percent of residents living in poverty**				
Intercept	−8.51	0.28	−37.50	<0.0001
Date	0.22	0.002	91.85	<0.0001
Percent in poverty	0.52	0.13	3.93	<0.0001
Date*percent in poverty	−0.03	0.003	−11.70	<0.0001
**Percent of residents living in deep poverty**				
Intercept	−8.52	0.22	−38.17	<0.0001
Date	0.21	0.002	99.37	<0.0001
Percent in deep poverty	0.32	0.13	2.50	0.012
Date*percent in deep poverty	-0.03	0.003	−9.33	<0.0001
**Social mobility index**				
Intercept	−8.43	0.23	−36.31	<0.0001
Date	0.19	0.002	94.61	<0.0001
Social mobility	−0.38	0.15	−2.48	0.013
Date*social mobility	−0.05	0.03	−1.67	0.344
**Percent of residents with less than high school diploma**				
Intercept	−7.20	0.37	−19.61	<0.0001
Date	0.12	0.005	23.42	<0.0001
<High school	−0.10	0.02	−4.79	<0.0001
Date* <high school	0.01	0.009	1.11	0.467
**Urban**				
Intercept	−8.72	0.0002	−58430.20	<0.0001
Date	0.23	0.0001	1624.90	<0.0001
Urban	2.75	0.0002	18449.50	<0.0001
Date*urban	−0.03	0.0001	−216.20	<0.0001
**Life expectancy**				
Intercept	−8.48	0.2367	−35.84	<0.0001
Date	0.25	0.003	81.13	<0.0001
Life expectancy	0.83	0.1344	6.205	<0.0001
Date*life expectancy	−0.04	0.22	−0.189	0.8810
**Percent low birth weight**				
Intercept	−8.60	0.222	−38.72	<0.0001
Date	0.22	0.002	107.12	<0.0001
Percent low birth weight	0.29	0.1175	2.43	0.0153
Date*percent low birth weight	0.07	0.003	24.25	<0.0001

**Table 12:** Mixed effects Poisson regression model simple slopes, standard errors, *t*, and *p*-values, for the relationship between poverty variables and the number of Covid-19 deaths.

**Date**	**Coefficient**	**Standard error**	***t***	***p*-value**	**Deaths**
**Percent living in poverty**					
March 22, 2020	1.12	0.90	1.23	0.12	504
April 1, 2020	1.88	0.88	2.14	0.03	4,778
**Percent living in deep poverty**					
March 22, 2020	0.92	0.91	1.01	0.17	
April 1, 2020	2.08	0.88	2.35	0.02	
**Urban**					
March 22, 2020	0.34	0.71	0.48	0.32	
April 1, 2020	2.51	1.37	1.83	0.048	
**Percent low birth weight**					
March 22, 2020	0.15	0.49	−0.55	0.39	
April 1, 2020	0.85	0.52	0.83	0.06	

## Discussion

The results of this study demonstrate that in the very early stages of the Covid-19 pandemic in the United States, counties with higher overall poverty (as reflected in the poverty index) had larger numbers of confirmed cases than did relatively more affluent counties. This trend changed over time. Through the months of February and March, 2020 there were more confirmed cases of the virus in poorer counties, but by April 1, 2020 the relationship had shifted so that the number of confirmed Covid-19 cases was greater in relatively less poor counties. When examining the relationships between specific facets of poverty and the number of confirmed Covid-19 cases, the results of this study demonstrated similar patterns for the percent of residents living in poverty and deep poverty, the social mobility index, life expectancy, percent low birth weight, and urban counties. Namely, early in the pandemic counties with higher rates of poverty, and deep poverty, as well as those with less social mobility, lower life expectancy, a higher percent of low birth weight babies, and more urban counties had greater numbers of confirmed Covid-19 cases, but this trend shifted by April 1, 2020 in much the same manner as for the overall poverty index.

Results for the number of deaths confirmed to be caused by Covid-19 demonstrated a pattern whereby the number of deaths was greater in areas of relatively greater poverty later in the pandemic. Furthermore, a larger number of deaths was associated with a larger percent of county residents living in poverty, living in deep poverty, a higher incidence of low weight births, and with the county being designated as urban. These trends were more pronounced on April 1, 2020 than in March. The estimated progression of the disease from infection to death of 16.1 days (Sanche et al., 2020) supports the temporal pattern of results for confirmed Covid-19 deaths and number of cases identified in this study. In short, individuals in relatively poor counties who were confirmed to have the virus in early to mid-March, 2020 would begin to die in late March and early April, given the mean 16.1 day period from infection to death, assuming that the presence of the virus was detected relatively early. However, it is also important to note that in some areas, testing was reserved for individuals who showed clear symptoms of Covid-19, and who thus may have had a relatively advanced illness. In such cases, the time between diagnosis and death could be much shorter.

Of particular interest in the current study was the relationship between poverty and the susceptibility of communities to contracting and dying from Covid-19. One somewhat unexpected result of this study was the apparent change over time in the relationship between poverty and the number of confirmed Covid-19 cases. There are a number of possible explanations that could be responsible for this shift. Certainly one possibility is that the disease simply became relatively less prevalent in these counties over time. Under that scenario, poor urban areas would see relatively fewer cases because the virus simply did not infect residents in those areas to the same extent that it did in relatively more affluent less urban communities. While certainly a theoretical possibility, evidence from the public health literature would suggest that the Covid-19 virus is highly infectious with a median *R*_0_ value of 5.7 (Sanche et al., 2020). This high level of contagion would suggest that within any community, the likelihood of the infection rate slowing down (even relatively) of its own accord this quickly seems unlikely.

A second possible explanation for the results presented above is that mitigation efforts such as sheltering in place and physical distancing had the desired effect more strongly in poorer, more urban counties than in relatively more affluent areas. However, there is some evidence that many jobs deemed to be essential, such as sanitation workers, operators of public transportation, and grocery store employees are relatively less well paid than those individuals who can work from home (Gray and Moore, 2020). In addition, lower income urban residents are more likely to use public transportation (Rachele et al., 2015), where exposure to the Covid-19 virus is more likely than would be the case in a private vehicle. Thus, while the possibility that mitigation efforts have been more effective in lower income more urban areas, there is some evidence to suggest that this may not in fact be the case.

A third possible explanation for the results presented above is that the limited testing resources available in the United States were diverted to relatively more affluent counties as the pandemic took hold in the nation. Prior research has shown that testing and treatment for influenza is relatively less available in less affluent communities (Ompad et al., 2007; Logan, 2009). Furthermore, preliminary findings reported by the Lerner Center for Public Health Promotion at Syracuse University (Monnat and Cheng, 2020) indicate that testing rates are lower in states with more black and poor residents. These findings, coupled with the acute shortage of tests that has beleaguered the entire American health care system from the beginning of the pandemic, would suggest the possibility of testing resources being less available in under-resourced communities compared to those with more financial means. Anecdotal reports in the news, while by no means definitive, do suggest a continuing serious Covid-19 problem in urban areas. Thus, it is possible that the trends evident early in the pandemic, in which more urban, less affluent counties had higher rates of Covid-19 cases, may actually be continuing but that testing resources are no longer available to confirm that the illnesses are in fact caused by the virus. This situation is exacerbated when individuals do not go the hospital when needed for fear of Covid-19 exposure while there, and by the fact that post-mortem testing is not done when individuals die at home, even when the symptoms at death would suggest the possibility of it being due to coronavirus (CNBC, 2020; New York Times, 2020).

### Limitations and Directions for Future Research

We hope that this study leads to further research with respect to the Covid-19 pandemic and poverty. Specifically, this work examined data that were collected in the initial stages of the outbreak in the United States. Undoubtedly, the counts of both cases and deaths will be refined over time, and a replication of this work should be conducted using such information. This is particularly true in the case of deaths, given the small numbers that were accounted to Covid-19 in the current dataset. It is possible that when mortality data are updated by public health officials these results could change.

An important limitation of the data used in the current study is that it reflects only those cases of Covid-19 that were reported to public health departments in the various counties. Thus, only people who were voluntarily tested, or who were referred to testing are included in the study. It is very likely that many individuals who were asymptomatic (or whose symptoms were extremely mild) but who did have the virus were not included in this dataset. This limitation would be especially pertinent in counties and states with limited testing capacities, where only the most seriously ill individuals would likely be tested.

### Implications

Given the available data, it is not possible to know definitively what factors led to the temporal differences in the relationship between poverty and the number of confirmed Covid-19 cases and deaths described above. However, given the patterns of disease transmission and level of contagion, it does seem reasonable to consider the possibility that a large part of the cause is due to a lack of testing capability in the United States. In that instance, it is possible, and given the progression of the disease, perhaps even likely that Covid-19 continued to appear in relatively high rates within poor urban counties, even as the number of available tests in those communities declined relative to more affluent areas. Only further investigation, and particularly an investigation into the allocation of scarce testing resources can answer this question with any certainty. In addition to this need for examining the equity in testing issue, the results of this study also demonstrate the need for the broader community to pay serious attention to public health emergencies that occur within every part of society. Even as the number of confirmed Covid-19 cases increased exponentially, and disproportionately within poorer urban counties in February and March, the U.S. government, as well as those of many states, did not engage in mitigation efforts, such as physical distancing. Indeed, most such orders were only given well after Covid-19 had become ensconced in these communities and begun to move into more affluent counties. Although impossible to know with certainty, it is not unreasonable to believe that had the larger community paid closer attention to the rising number of cases in less affluent areas, and done more to help the residents therein, that the severity of the pandemic both in those counties, and in the nation more broadly could have been ameliorated.

These results also suggest that under-resourced workers in fields that have been deemed essential (e.g., public sanitation, grocery employees, delivery services) and who thus may be at particular risk may not have equal access to testing for the virus. These workers, though at elevated risk, may be without the ability to quarantine away from their families in the same manner as do health care workers, another group at higher risk for exposure to the coronavirus. Given that the limited testing resources have now been diverted to health care workers (NBC News, 2020), certainly with good reason, this problem of potential underreporting of Covid-19 cases in under-resourced communities may continue to be a serious problem.

## Data Availability Statement

The datasets presented in this study can be found in online repositories. The names of the repository/repositories and accession number(s) can be found in the article/supplementary material.

## Author Contributions

WF ran data analysis and wrote sections of the text. MH wrote sections of the text, and provided interpretation of the results. All authors contributed to the article and approved the submitted version.

## Conflict of Interest

The authors declare that the research was conducted in the absence of any commercial or financial relationships that could be construed as a potential conflict of interest.
